# A comprehensive expression analysis of the MIA gene family in malignancies: MIA gene family members are novel, useful markers of esophageal, lung, and cervical squamous cell carcinoma

**DOI:** 10.18632/oncotarget.9082

**Published:** 2016-04-28

**Authors:** Tomonori Sasahira, Tadaaki Kirita, Yukiko Nishiguchi, Miyako Kurihara, Chie Nakashima, Anja Katrin Bosserhoff, Hiroki Kuniyasu

**Affiliations:** ^1^ Department of Molecular Pathology, Nara Medical University, Kashihara, Nara, Japan; ^2^ Department of Oral and Maxillofacial Surgery, Nara Medical University, Kashihara, Japan; ^3^ Institute for Biochemistry, Friedrich-Alexander University Erlangen-Nürnberg, Erlangen, Germany

**Keywords:** MIA, MIA2, TANGO, SCC

## Abstract

Melanoma inhibitory activity *(MIA)* gene family members include MIA, MIA2, and Transport and Golgi organization protein 1 (TANGO). Although *MIA* gene family members have several tumor-related functions, their detailed roles in malignancies remain poorly elucidated. In this study, 477 tumor specimens were subjected to immunohistochemical screening to evaluate *MIA* gene family expression. For a validation analysis, we also examined the association between *MIA* gene family expression and clinicopathological factors in 66 cases of esophageal cancer, 145 cases of lung cancer, and 126 cases of cervical cancer. The frequency of *MIA* gene family expression was higher among squamous cell carcinomas than among other tumor types subjected to screening. In the validation analysis, *MIA* gene family staining was observed frequently in esophageal and lung cancers associated with nodal and/or distant metastasis. In cervical cancers, MIA and TANGO immunostaining also correlated with tumor progression and metastasis. Furthermore, MIA2 expression levels in invasive cervical cancer were upregulated relative to those in cervical intraepithelial neoplasia 3. A disease-free survival analysis revealed that MIA-, MIA2, or TANGO-positive patients had a significantly shorter disease-free survival than did those patients who were negative. Our results suggest that MIA, MIA2, and TANGO may be useful diagnostic and therapeutic molecular targets in human malignancies.

## INTRODUCTION

An estimated 455 800 and 527 600 new cases and 400 200 and 265 700 deaths related to esophageal and cervical cancer, respectively, have been reported worldwide [1]. Notably, postoperative recurrences occur in approximately half of all patients with esophageal squamous cell carcinoma (ESCC) [2], and it is the third leading cause of cancer deaths among women in developing countries [1]. Additionally, an estimated 1.8 million new lung cancer cases have occurred worldwide, accounting for approximately 13% of all cancers [1]. Therefore, the early detection of such malignancies is urgently necessary.

The melanoma inhibitory activity (*MIA*) gene family includes MIA, MIA2, Transport and Golgi organization protein 1 (TANGO), and otoraplin (OTOR). Members of this family share 34%–45% amino acid homology and 47%–59% cDNA sequence homology and feature a highly conserved SH3-like domain and hydrophobic N-terminal secretory signal sequences [3–6]. Although OTOR expression is highly restricted to healthy eyes, cochlea, and cartilage [7], other members of the *MIA* gene family have several tumor-related functions. MIA expression correlates with cancer cell detachment, migration, invasion, and apoptotic repression and is accordingly related to malignant tumor progression, metastasis, and poor prognosis [8–12]. MIA2 is induced in liver fibrosis or cirrhosis by activating transforming growth factor-beta (TGF-β) signaling [13, 14] and serves as a tumor suppressor in liver cancers following the loss of hepatocyte nuclear factor-1 (HNF-1) expression [15]. However, wild-type MIA2 promotes the loss of chemosensitivity in pancreatic cancers, thus worsening an already poor prognosis [16]. Regarding other *MIA* gene family members, TANGO has been suggested as a tumor suppressor in malignant melanoma, colorectal cancer, and hepatoma [4, 6]. In summary, the functions of *MIA* gene family members in malignancies have not been well documented.

We previously reported that *MIA* gene family members act as oncogenes in oral squamous cell carcinoma (OSCC) [17–20]. For example, MIA expression is enhanced by high-mobility group box 1 (HMGB1) nuclear factor kappa B (NFkB) p65 complexes that bind to the *MIA* promoter region, thus promoting tumor progression, nodal metastasis, a worse prognosis, angiogenesis, and lymphangiogenesis through the upregulation of vascular endothelial growth factor (VEGF)-A, VEGF-C, and VEGF-D expression [17, 18]. MIA2 activates MAPK p38, c-Jun N-terminal kinase (JNK), and VEGF family members through its receptors, integrin α_4_β_1_ and α_5_β_1_ [19]. MIA2 expression is also associated with local expansion, nodal metastasis, and inhibited host anti-cancer immunity and apoptosis in OSCC [19]. Furthermore, TANGO promotes angiogenesis and lymphangiogenesis by upregulating platelet-derived growth factor beta polypeptide (PDGFB) and neuropilin 2 in OSCC [20].

Tumor biomarkers have been classified as screening (used diagnostically to identify patients), staging (used to stage disease), prognostic (used to predict outcome), and predictive and monitoring markers (used to speculate and observe clinical responses to any treatment) [21, 22]. Cancer biomarkers must also satisfy the following conditions: (1) the transition can be objectively determined the quality; (2) must be measureable in small sample amounts; (3) must be altered in tumors but not in normal tissues; and (4) must be altered at an early phase of cancer development [22, 23]. However, the role of MIA gene family as tumor markers in various human malignancies remains controversial. The purpose of this study was to investigate the usefulness of *MIA* gene family as novel tumor markers in various human neoplastic specimens, including ESCC, lung cancer, and cervical cancer.

## RESULTS

### Screening for MIA gene family expression in human tumors

**Figure 1:**
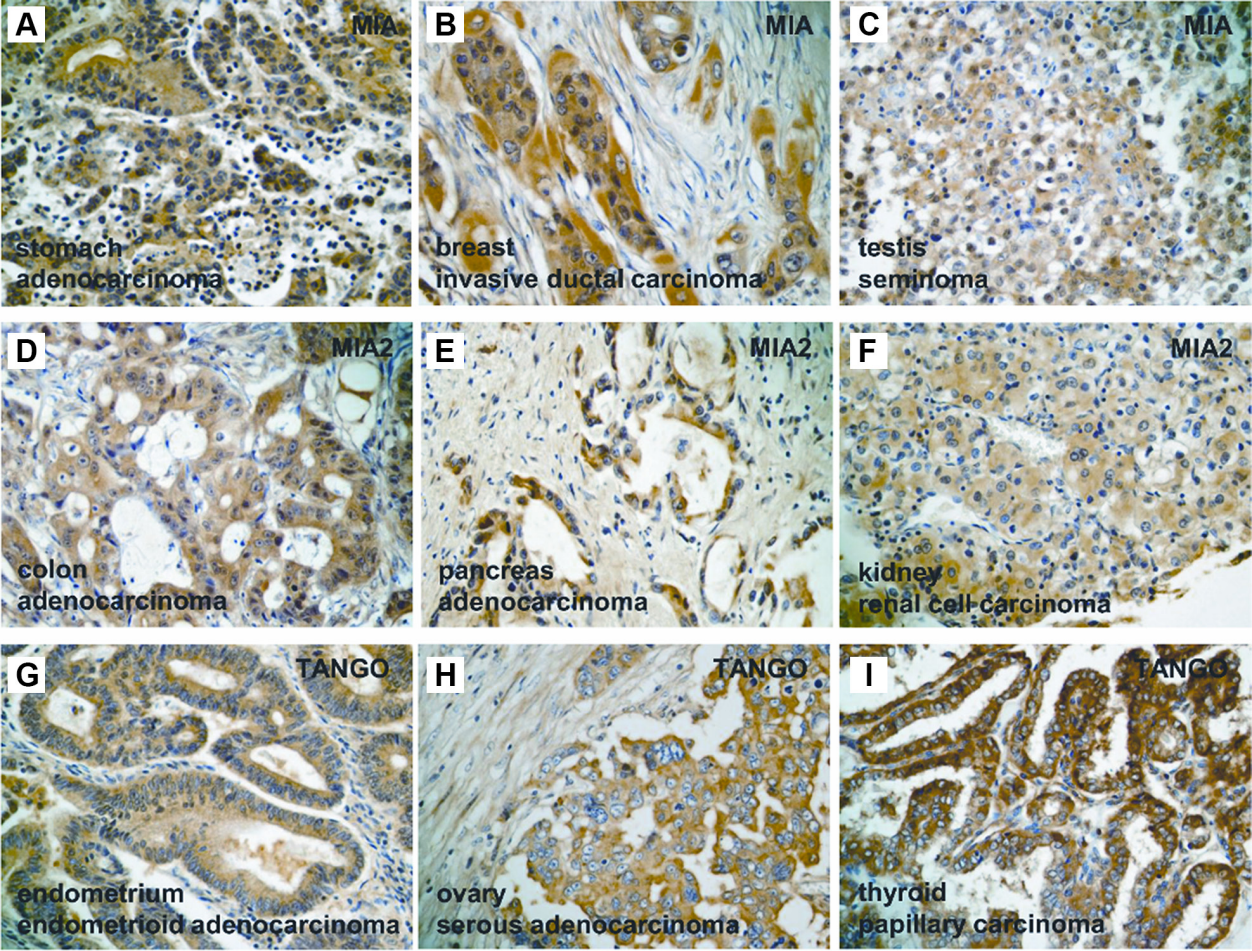
Expression of *MIA* gene family in human tumors (**A–C**) Immunostaining of MIA in human tumors. Cytoplasmic expression of MIA was detected in chondrosarcoma of the bone (A), invasive ductal carcinoma of the breast (B), and seminoma of the testis (C). (**D–F**) Immunostaining of MIA2 in human tumors. Cytoplasmic localization of MIA2 was found in colonic adenocarcinoma (D), pancreatic adenocarcinoma (E), and renal cell carcinoma (F). (**G–I**) Immunostaining of TANGO in human tumors. Cytoplasmic staining of TANGO was observed in endometrial endometriod adenocarcinoma (G), ovarian serous cystadenocarcinoma (H), and papillary carcinoma of the thyroid (I). Original magnification, 400 fold.

We initially used immunohistochemistry to examine the expression of *MIA* gene family members in 477 cases of different tumors. The specificity of the antibodies for *MIA* gene family was confirmed by Western blotting with recombinant proteins (data not shown). These results are summarized in Table 1. Briefly, higher MIA, MIA2, and TANGO expression levels were observed in 80 (16.8%), 67 (14.1%), and 76 (15.9%) of these cases, respectively. All immunopositive cases exhibited cytoplasmic *MIA* gene family staining. Several representative images of *MIA* gene family immunostaining in tumors are shown in Figure 1A to 1I.

**Table 1:** Expression of MIA gene family in human tumors

		Number of overexpression cases		
Organ and histology	Number of cases	MIA	MIA2	TANGO
Head and neck (oral cavity, larynx, pharynx)				
Squamous cell carcinoma	30	13 (43.3%)	12 (40%)	10 (33.3%)
Others	6	0 (0%)	0 (0%)	0 (0%)
Salivary gland				
Acinic cell carcinoma	3	0 (0%)	0 (0%)	1 (33.3%)
Adenoid cystic carcinoma	3	1 (33.3%)	1 (33.3%)	1 (33.3%)
Mucoepidermoid carcinoma	2	0 (0%)	0 (0%)	0 (0%)
Others	7	1 (14.3%)	0 (0%)	0 (0%)
Esophagus				
Squamous cell carcinoma	10	4 (40%)	4 (40%)	4 (40%)
Stomach				
Adenocarcinoma	35	10 (28.6%)	5 (14.3%)	6 (17.1%)
Small intestine				
Gastrointestinal stromal tumor	5	0 (0%)	0 (0%)	0 (0%)
Colorectum				
Adenocarcinoma	35	11 (31.4%)	6 (17.1%)	8 (22.9%)
Liver				
Hepatocellular carcinoma	16	0 (0%)	7 (43.8%)	3 (18.8%)
Biliary system				
Adenocarcinoma	10	1 (10%)	2 (20%)	1 (10%)
Pancreas				
Adenocarcinoma	5	0 (0%)	2 (40%)	1 (20%)
Lung				
Squamous cell carcinoma	18	6 (33.3%)	5 (27.8%)	6 (33.3%)
Adenocarcinoma	11	2 (18.2%)	2 (18.2%)	2 (18.2%)
Others	4	0(0%)	0 (0%)	0 (0%)
Soft tissue				
Liposarcoma	12	0 (0%)	0 (0%)	0 (0%)
Undifferentiated pleomorphic sarcoma	4	0 (0%)	0 (0%)	0 (0%)
Others	9	0 (0%)	0 (0%)	0 (0%)
Bone				
Chondrosarcoma	8	4 (50%)	0 (0%)	0 (0%)
Osteosarcoma	3	0 (0%)	0 (0%)	0 (0%)
Others	5	0 (0%)	0 (0%)	0 (0%)
Skin				
Malignant melanoma	10	6 (60%)	2 (20%)	2 (20%)
Squamous cell carcinoma	3	1 (33.3%)	1 (33.3%)	1 (33.3%)
Breast				
Invasive ductal carcinoma	7	2 (28.6%)	0 (0%)	3 (42.9%)
Invasive lobular carcinoma	4	0 (0%)	1 (25%)	1 (25%)
Others	8	0 (0%)	0 (0%)	0 (0%)
Uterine cervix				
Squamous cell carcinoma	21	8 (38.1%)	6 (28.6%)	7 (33.3%)
Others	5	0 (0%)	0 (0%)	0 (0%)
Endometrium				
Endometrioid adenocarcinoma	11	0 (0%)	3 (27.3%)	3 (27.3%)
Others	1	0 (0%)	0 (0%)	0 (0%)
Ovary				
Serous adenocarcinoma	11	2 (18.2%)	1 (9.1%)	2 (18.2%)
Mucinous adenocarcinoma	8	0 (0%)	1 (12.5%)	1 (12.5%)
Endometrioid adenocarcinoma	5	1 (20%)	0 (0%)	1 (20%)
Clear cell carcinoma	4	0 (0%)	0 (0%)	0 (0%)
Dysgerminoma	4	0 (0%)	0 (0%)	1 (25%)
Others	15	1 (6.7%)	0 (0%)	0 (0%)
Prostate				
Adenocarcinoma	6	2 (33.3%)	0 (0%)	2 (33.3%)
Testis				
Seminoma	8	2 (25%)	0 (0%)	2 (25%)
Others	3	0 (0%)	0 (0%)	0 (0%)
Urinary bladder				
Urothelial carcinoma	17	2 (11.8%)	1 (5.9%)	3 (17.7%)
Kidney				
Renal cell carcinoma	13	0 (0%)	5 (38.5%)	0 (0%)
Others	3	0 (0%)	0 (0%)	0 (0%)
Thyroid				
Papillary carcinoma	12	0 (0%)	0 (0%)	4 (33.3%)
Others	3	0 (0%)	0 (0%)	0 (0%)
Adrenal gland				
Cortical carcinoma	10	0 (0%)	0 (0%)	1 (10%)
Pheochromocytoma	3	0 (0%)	0 (0%)	0 (0%)
Others	2	0 (0%)	0 (0%)	0 (0%)
Lymphoid tissue				
Malignant lymphoma	20	0 (0%)	0 (0%)	0 (0%)
Other tissues	19	0 (0%)	0 (0%)	0 (0%)

MIA overexpression was observed in 13 of 30 head and neck SCCs (43.3%), 4 of 10 ESCCs (40%), 10 of 35 gastric adenocarcinomas (28.6%) (Figure 1A), 11 of 35 colorectal adenocarcinomas (31.4%), 6 of 18 lung SCCs (33.3%), 1 of 3 cutaneous SCCs (33.3%), 2 of 7 mammary invasive ductal carcinomas (28.6%) (Figure 1B), 8 of 21 cervical SCCs (38.1%), 2 of 6 prostatic adenocarcinomas (33.3%), and 2 of 8 testicular seminomas (25%) (Figure 1C). Twelve of the 30 head and neck SCCs (40%), 3 of 10 ESCCs (30%), 7 of 16 hepatocellular carcinomas (43.8%), 2 of 5 pancreatic adenocarcinomas (40%) (Figure 1E), 5 of 18 lung SCCs (27.8%), 2 of 10 cutaneous malignant melanomas (20%), 1 of 3 cutaneous SCCs (33.3%), 6 of 21 cervical SCCs (28.6%), 3 of 11 endometrial endometrioid adenocarcinomas (27.3%), and 5 of 13 renal cell carcinoma (38.5%) (Figure 1F) exhibited excessive MIA2 immunoreactivity. Elevated TANGO expression levels were detected in 10 of 30 head and neck SCCs (33.3%), 3 of 10 ESCCs (30%), 8 of 35 colorectal adenocarcinomas (22.9%), 1 of 5 pancreatic adenocarcinomas (20%), 6 of 18 lung SCCs (33.3%), 2 of 10 cutaneous malignant melanomas (20%), 1 of 3 cutaneous SCCs (33.3%), 3 of 7 mammary invasive ductal carcinomas (32.9%), 7 of 21 cervical SCCs (33.3%), 3 of 11 endometrial endometrioid adenocarcinomas (27.3%) (Figure 1G), 2 of 6 prostatic adenocarcinomas (33.3%), 2 of 8 testicular seminomas (25%), and 4 of 12 papillary thyroid carcinomas (33.3%) (Figure 1I). MIA2 and TANGO immunostaining in a colonic adenocarcinoma and ovarian serous adenocarcinoma are shown in Figure 1D and 1H, respectively. Overall, SCCs were more likely to express *MIA* gene family members. Detailed MIA, MIA2, and TANGO immunohistochemistry results in other human malignant tumors are presented in Table 1.

We formerly reported that MIA, MIA2, and TANGO act as oncogenes in OSCC [17–20]; however, the significance of *MIA* gene family expression in SCCs of other organs, such as the esophagus, cervix, and lung, has not been clarified. Accordingly, we next inspected the relationship between *MIA* gene family immunostaining and clinicopathological features in those malignancies.

### Relationship MIA gene family expression and clinicopathological parameters in esophageal cancers

**Figure 2:**
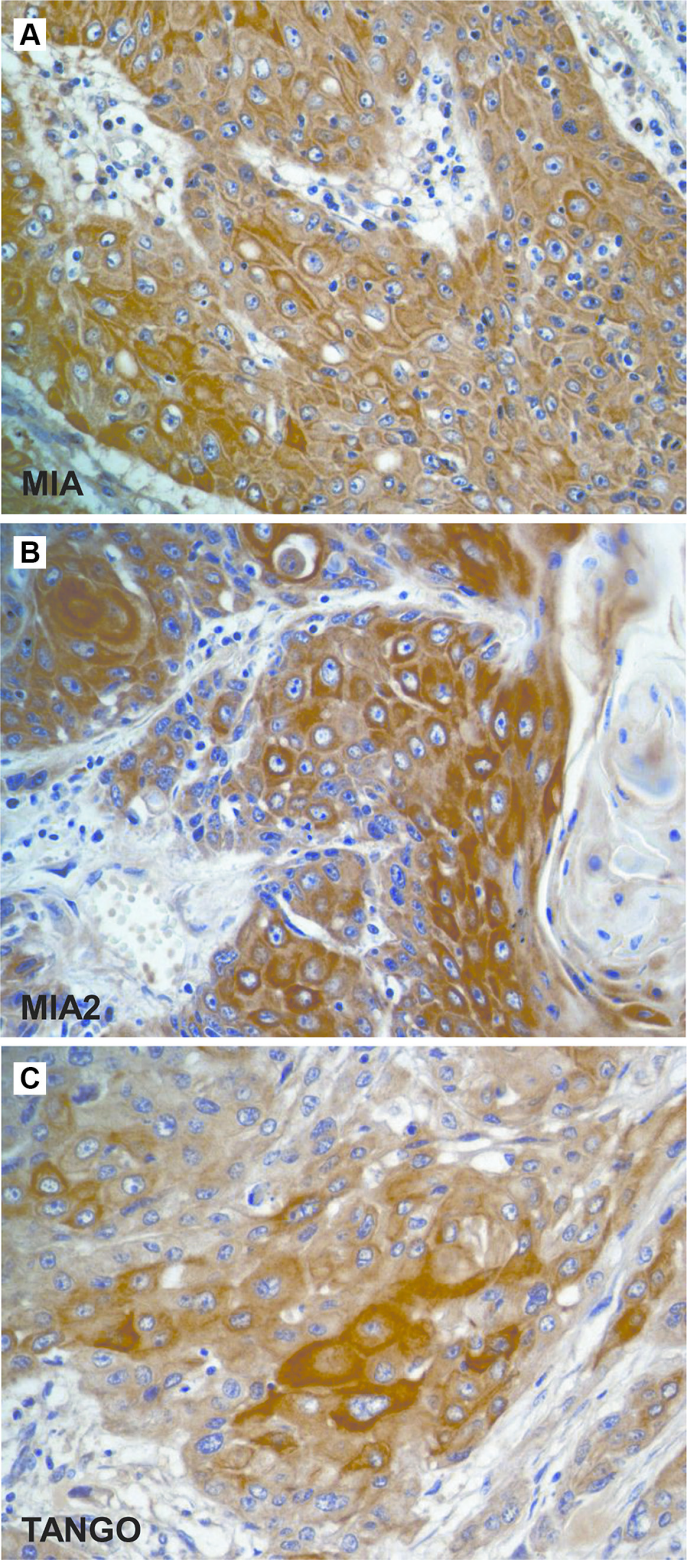
Immunostaining of the *MIA* gene family in esophageal cancer (**A–C**) Immunohistochemistry of the *MIA* gene family in human ESCC. Immunostaining of MIA (A), MIA2 (B), and TANGO (C) was detected in the cytoplasm of ESCC cells. Original magnification, 400 fold. ESCC: esophageal squamous cell carcinoma.

Compared with non-tumor esophageal epithelium, ESCC tissues were more likely to exhibit subcellular *MIA* gene family expression. MIA, MIA2, and TANGO overexpression was detected in 33.3% (22/66), 30.3% (20/66), and 27.3% (18/66) of ESCC cases, respectively (Figure 2A–2C). Further, co-expression rate of MIA and MIA2, MIA and TANGO, MIA2 and TANGO, and all molecules were 15.2% (10/66), 16.7% (11/66), 13.6% (9/66), and 9.1% (6/66), respectively. The correlations between *MIA* gene family expression and clinicopathological parameters are summarized in Table 2. MIA upregulation correlated only with nodal metastasis, and 15 of 32 (46.9%) patients with nodal metastasis exhibited MIA immunopositivity (*P* = 0.0362). On the other hand, MIA2 expression correlated significantly with the clinical stage (*P* = 0.0026), local tumor cell progression (T classification; *P* = 0.0076), and nodal metastasis (*P* = 0.0069). Furthermore, TANGO expression was observed in 14 of 32 (43.8%) and 9 of 13 (69.2%) cases involving nodal or distant metastasis, respectively, with lower rates among patients with no nodal progression (4/34, 11.8%; *P* = 0.0053) or distant metastasis (9/53, 17%; *P* = 0.0005). No strong relationships of MIA, MIA2, or TANGO expression with other clinicopathological characteristics were found in ESCC cases.

**Table 2:** Relationship between expression of MIA gene family and clinicopathological characteristics in ESCC

	MIA		MIA2		TANGO	
Parameters	negative	positive	negative	positive	negative	positive
Gender						
Male	31	18	37	12	34	15
Female	13	4	9	8	14	3
*P* value	0.3836		0.1244		0.3614	
Age						
< −65	14	8	13	9	17	5
> 65	30	14	33	11	31	13
*P* value	0.7848		0.2565		0.7702	
Alcohol intake						
Habitual drinking	24	16	25	15	26	14
Social drinking	14	6	16	4	16	4
No drinking	6	0	5	1	6	0
*P* value	0.1423		0.2844		0.1362	
Histological differentiation*						
Well	23	14	25	12	26	11
Mod, Por	21	8	21	8	22	7
*P* value	0.4381		0.7894		0.7817	
Clinical stage						
I–II	25	9	28	6	28	6
III–IV	19	13	18	14	20	12
*P* value	0.2978		0.0317		0.0983	
T classification						
T1–2	22	8	26	4	24	6
T3–4	22	14	20	16	24	12
*P* value	0.4320		0.0076		0.2748	
Nodal metastasis						
Negative	27	7	29	5	30	4
Positive	17	15	17	15	18	14
P value	0.0362	0.0069	0.0053			
Distant metastasis						
Negative	37	16	38	15	44	9
Positive	7	6	8	5	4	9
*P* value	0.3313		0.5117		0.0005	

Relationship between expression of MIA gene family and parameters were calculated by Fischer's exact test. T classification and clinical stage were classified according to the TNM classification.

* Histological differentiation: Well, well-differentiated squamous cell carcinoma; Mod, moderately differentiated squamous cell carcinoma; Por, poorly differentiated squamous cell carcinoma.

### Association between MIA gene family expression and clinicopathological characteristics in lung cancers

**Figure 3:**
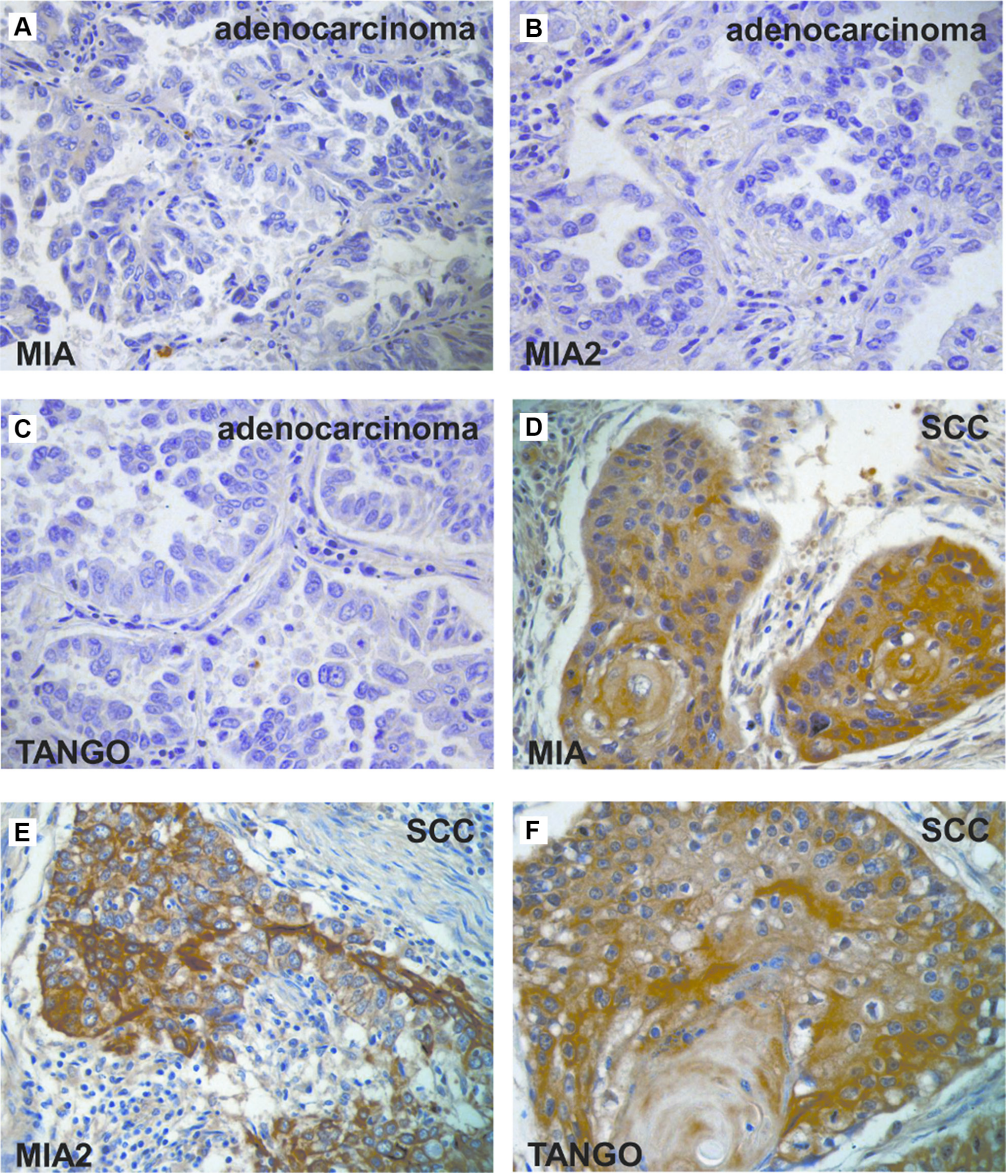
Expression of the *MIA* gene family in lung cancer (**A–C**) Immunohistochemical analysis of the *MIA* gene family in adenocarcinoma. Expression of MIA (A), MIA2 (B), and TANGO (C) was not detected in lung adenocarcinoma. (**D–F**) Immunohistochemistry of the *MIA* gene family in SCC. Cytoplasmic localization of MIA (D), MIA2 (e), and TANGO (F) was observed in lung SCC. Original magnification, 400 fold. SCC: squamous cell carcinoma.

A summary of the results pertaining to lung cancer is shown in Table 3. Non-cancerous lungs did not overexpress *MIA* gene family members; in contrast, cytoplasmic MIA, MIA2, and TANGO expression was found in 49 (33.8%), 45 (31%) and 47 (32.4%) of the 145 cases, respectively (Figure 3A–3F). Additionally, co-expression rate of MIA and MIA2, MIA and TANGO, MIA2 and TANGO, and all molecules were 24.8% (36/145), 17.2% (25/145), 19.3% (28/145), and 13.8% (20/145), respectively. Strong MIA, MIA2, and TANGO immunoreactivity levels were detected in 41% (43/105), 37.1% (39/105), and 40% (42/105) of SCCs, respectively; notably, the MIA, MIA2, and TANGO expression frequencies in adenocarcinomas, small cell carcinomas, and LCNECs were significantly lower than those in SCCs (Figure 3A–3F; *P* = 0.0307, *P* = 0.0453, and *P* = 0.0173, respectively). Higher MIA (21/36, 58.3%), MIA2 (19/36, 52.8%), and TANGO expression levels (21/36, 58.3%) were observed in cases with nodal metastasis relative to those without lymph node involvement (*P* = 0.0005, *P* = 0.0018, and *P* = 0.0004, respectively). MIA and MIA2 expression also correlated with distant metastasis; 11 of 17 (64.7%) patients with distant metastases exhibited MIA and MIA2 immunopositivity (*P* = 0.0063 and *P* = 0.0036, respectively). Moreover, TANGO expression correlated significantly with the T classification (*P* = 0.0177). There were no significant relationships between MIA gene family expression and other clinicopathological parameters in lung cancers.

**Table 3:** Relationship between expression of MIA gene family and clinicopathological characteristics in lung cancer

	MIA		MIA2		TANGO	
Parameters	negative	positive	negative	positive	negative	positive
Gender						
Male	69	38	71	36	69	38
Female	27	11	29	9	29	9
*P* value	0.5512		0.3101		0.2276	
Age						
< −65	47	20	49	18	47	20
> 65	49	29	51	27	51	27
*P* value	0.3827		0.3696		0.5957	
Smoking habit						
Yes	63	39	67	35	64	38
No	33	10	33	10	34	9
*P* value	0.0879		0.2394		0.0796	
Histology *						
SCC	62	43	66	39	63	42
Adeno	21	4	20	5	22	3
Small	9	1	10	0	9	1
LCNEC	4	1	4	1	4	1
*P* value	0.0307		0.0453		0.0173	
Clinical stage						
I	18	13	24	8	26	6
II	43	20	42	21	45	18
III–IV	34	16	34	16	27	23
*P* value	0.5766		0.6971		0.0252	
T classification						
T1	19	13	24	8	26	6
T2	38	18	36	20	41	15
T3–4	39	18	40	17	31	26
*P* value	0.6503		0.5609		0.0177	
Nodal metastasis						
Negative	81	28	83	26	83	26
Positive	15	21	17	19	15	21
*P* value	0.0005		0.0018		0.0004	
Distant metastasis						
Negative	90	38	94	34	87	41
Positive	6	11	6	11	11	6
*P* value	0.0063		0.0036		0.7876	

Relationship between expression of MIA gene family and parameters were calculated by Fischer's exact test or chi-square test. T classification and clinical stage were classified according to the TNM classification.

* Histology: SCC, squamous cell carcinoma; adeno, adenocarcinoma; small, small cell carcinoma; LCNEC, large cell neuroendocrine cell carcinoma.

### Correlation between MIA gene family expression and clinicopathological features in cervical cancers

**Figure 4:**
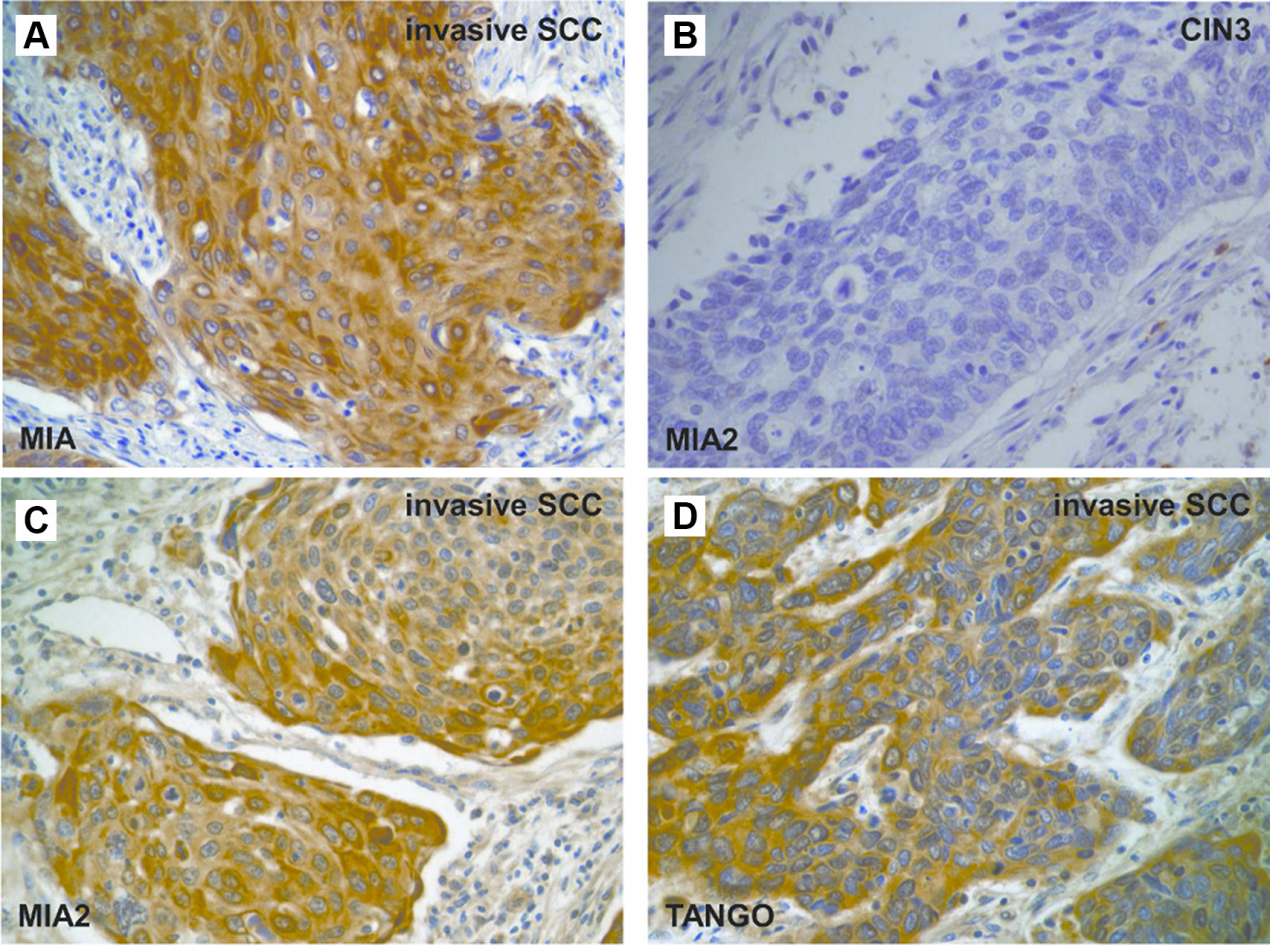
Expression of the *MIA* gene family in cervical cancer (**A, C–D**) Immunostaining of the *MIA* gene family in invasive SCC. MIA (A), MIA2 (C), and TANGO (D) expression were observed in the cytoplasm of invasive cervical SCC. (**B**) Cases with CIN3 showed no immunoreactivity of MIA2. Original magnification, 400 fold. SCC: squamous cell carcinoma.

The detailed immunohistochemical results of the 126 cervical cancers selected for the second cohort are summarized in Table 4. Non-tumor cervical mucosal samples had negative or very weak *MIA* gene family expression, whereas 34.1% (43/126), 31% (39/126), and 30.2% (38/126) of cervical cancer cases exhibited cytoplasmic MIA, MIA2, and TANGO staining, respectively (Figure 4A–4D). Moreover, co-expression rate of MIA and MIA2, MIA and TANGO, MIA2 and TANGO, and all molecules were 13.5% (17/126), 10.3% (13/126), 12.7% (16/126), and 7.9% (10/126), respectively. In cervical cancers, increased MIA expression correlated strongly with the clinical stage (*P* = 0.0177), T classification (*P* = 0.0185), and lymph node metastasis (*P* = 0.0477). No obvious correlations were identified between the level of MIA and TANGO expression and tumor histological type; in contrast, the MIA2 expression levels increased from CIN3 (1/15, 6.7%) to invasive SCC (38/111, 34.2%; *P* = 0.0361; Figure 4B, 4C). TANGO overexpression was more frequent in cases with nodal metastasis (7/11, 63.6%) or distant metastasis than in those without metastasis (31/115, 27%; *P* = 0.0175). In addition, TANGO immunostaining was observed in 83.3% (5/6) of cases with distant metastasis and only 27.5% (33/120) of cases without distant metastasis (*P* = 0.0095). No significant difference was observed between *MIA* gene family expression and other clinicopathological features in cervical cancers.

**Table 4:** Relationship between expression of MIA gene family and clinicopathological characteristics in cervical cancer

	MIA		MIA2		TANGO	
Parameters	negative	positive	negative	positive	negative	positive
Age						
< −60	38	14	37	15	39	13
> 60	45	29	50	24	49	25
*P* value	0.1835		0.6999		0.3287	
Histology*						
CIN3	11	4	14	1	10	5
SCC	72	39	73	38	78	33
*P* value	0.5777		0.0361		0.7704	
HPV16 and/or 18						
Positive	53	33	56	30	57	29
Negative	33	10	31	9	31	9
*P* value	0.1131		0.2147		0.2192	
Clinical stage						
0–I	35	14	39	10	34	15
II	43	19	38	24	46	16
III–IV	5	10	10	5	8	7
*P* value	0.0177		0.1145		0.2861	
T classification						
Tis-T1	34	14	38	10	33	15
T2	45	20	39	26	49	16
T3–4	4	9	10	3	6	7
*P* value	0.0185		0.0755		0.1087	
Nodal metastasis						
Negative	79	36	81	34	84	31
Positive	4	7	6	5	4	7
*P* value	0.0477		0.3135		0.0175	
Distant metastasis						
Negative	80	40	84	36	87	33
Positive	3	3	3	3	1	5
*P* value	0.4096		0.3725		0.0095	

Relationship between expression of MIA genefamily and parameters were calculated by Fischer's exact test or chi-square test. T classification and clinical stage were classified according to the TNM classification.

* Histology: CIN3, cervical intraepithelial neoplasia 3; SCC, squamous cell carcinoma

### Gene expression of MIA gene family and secretion of MIA in esophageal, lung, and cervical cancers

**Figure 5:**
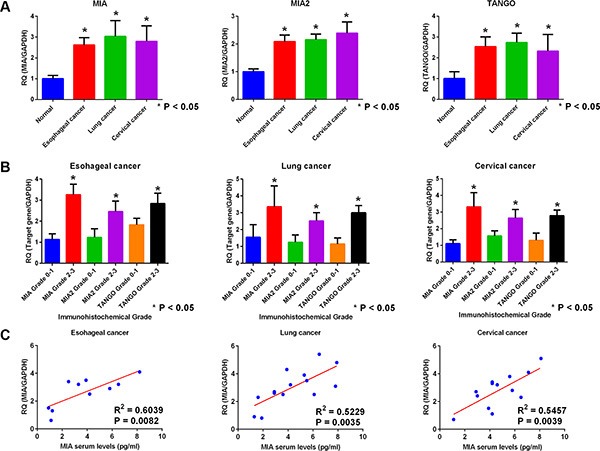
Gene expression and secretion of the *MIA* gene family in esophageal, lung, and cervical cancer (**A**) Expression of *MIA*, *MIA2*, and *TANGO* in esophageal, lung, and cervical cancer by real-time RT-PCR. Normal esophageal, lung, and cervical tissues were set as 1. Expression levels of MIA, MIA2, and TANGO in malignancies were higher than normal tissues. (**B**) Association of MIA, MIA2, or TANGO expression with immunohistochemical grade in cases with esophageal, lung, and cervical cancer. In malignancies, expression levels of each genes were well correlated with immunohistochemical grade. (**C**) Comparison of MIA levels with serum and primary tumor. Tumor expression levels of *MIA* were significantly correlated with those of serum secretion levels in esophageal, lung, and cervical cancer. Error bar, standard deviation (SD). RQ; relative quantification.

Next, we verified the expression of *MIA* family genes in cases with esophageal, lung, and cervical cancers. In malignancies, expression levels of *MIA*, *MIA2*, and *TANGO* were significantly higher than in non-tumorous specimens (Figure 5A). Moreover, the expression of *MIA* family genes was significantly associated with immunohistochemical grade in esophageal, lung, and cervical cancers (Figure 5B). Expression levels of *MIA* gene family in primary tumor and metastatic sites remained unchanged (data not shown).

Next, *MIA* gene family expression levels were compared between serum samples and tumor specimens. Serum secretion levels of MIA measured by enzyme-linked immunosorbent assay (ELISA) were well correlated with those of tumor expression levels quantified by quantitative (qRT-PCR) (Figure 5C).

### Disease free survival analysis of esophageal, lung, and cervical cancers

**Figure 6:**
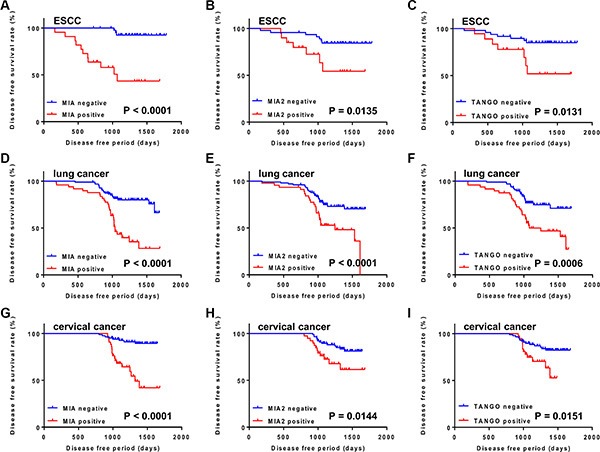
Disease free survival curves of cancer patients, as calculated by Kaplan–Meier method (**A–C**) Disease free survival curves in ESCC cases. Disease free survival period was significantly shorter in patients with MIA (A), MIA2 (B), and TANGO (C) expression than in those with no expression. (**D–F**) Disease free survival curves in lung cancer cases. Cases with expression of MIA (D), MIA2 (E), and TANGO (F) had significantly worse prognosis than those with negative expression. (**G–I**) Disease free survival curves in cervical cancer cases. MIA (G), MIA2 (H), and TANGO (I) expression cases were correlated with poor prognosis. The *P*-value was calculated using the log-rank test.

Finally, we conducted a Kaplan–Meier survival analysis. We found that patients with ESCC whose samples exhibited positive MIA, MIA2, and TANGO immunostaining had significantly shorter disease-free survival intervals, compared to patients with negative expression (*P* < 0.0001, *P* = 0.0135, and *P* = 0.0131, respectively; Figure 6A–6C). Among patients with lung cancer, those with MIA, MIA2, and TANGO-positive samples had a significantly worse disease free survival than did those with negative samples (*P* < 0.0001, *P* < 0.0001, and *P* = 0.0006, respectively; Figure 6D–6F). Furthermore, MIA (*P* < 0.0001), MIA2 (*P* = 0.0144), and TANGO expression (*P* = 0.0151) were associated with a poor prognosis among patients with cervical cancer (Figure 6G–6I).

## DISCUSSION

Although *MIA* gene family members serve several tumor-related functions, to our knowledge, this is the first report to subject a variety of human malignancies to semi-comprehensive immunohistochemical *MIA* gene family expression profiling. In this investigation, we found that *MIA* gene family members are frequently expressed in several types of human tumors, including SCCs. We also confirmed the significance of *MIA* gene family expression in ESCC, lung cancer, and cervical cancer. In particular, cases of ESCC and lung cancer with nodal and/or distant metastases were frequently positive for *MIA* gene family expression; similarly, lung SCCs were frequently positive for these proteins. In addition, *MIA* gene family expression was also associated with a poor prognosis among cancer patients. However, further research is needed to determine the association between *MIA* gene family expression and clinicopathological significance in tumors. TMA has recently become as powerful tool for large-scale expression analysis. TMA immunohistochemistry is a valuable high-throughput analysis technique because it eliminates technical variations among cases by subjecting all tissue cores to equal immunostaining conditions [24]. Additional immunohistochemical analyses of *MIA* gene family expression using large numbers of TMA slides will likely be effective.

In the present study, we have demonstrated the expression of MIA in previously uninvestigated tumors. Notably, MIA promotes cell detachment, migration, and invasion and suppresses cancer cell apoptosis and lymphokine activated killer cell (LAK) infiltration. In addition, MIA is a ligand for the cell surface receptors integrin α_4_β_1_/α_5_β_1_ and binds to fibronectin via SH3 domain-like structures to inhibit cell-to-stromal adhesion [9, 25, 26]. In a previous report, we described the activation of MIA via interactions of intracellular HMGB1 with NFkBp65 and observed the strong implication of MIA in tumor progression and nodal metastasis through the induction of angiogenesis and lymphangiogenesis in OSCC [17, 18]. MIA expression has also been observed in malignant melanoma, gastric cancer, pancreatic cancer, breast cancer, chondrosarcoma, glioma, and OSCC [7–12, 17, 18, 27–30].

Several reports have revealed that MIA2 and TANGO can act as tumor suppressors [4–6, 15]; it is therefore interesting that according to our current results and previous findings, MIA2 and TANGO might behave as proto-oncogenes in SCCs of the esophagus, lung, and cervix [19, 20]. These potential oncogenic roles might depend on receptor-related signaling differences. Indeed, we revealed that signaling through the MIA2-integrin α_5_β_1_-JNK pathway promotes apoptosis, whereas signaling through the MIA2-integrin α_4_β_1_-MAPK p38 pathway suppresses apoptosis [19]. Furthermore, MIA2 inhibits lymphocyte infiltration into tumors by binding integrin α_4_, thus dysregulating the host immune system [19]. Similar to MIA, MIA2 might also interact with fibronectin, which induces T lymphocyte chemotaxis when combined with stromal cell-derived factor 1α [31]; therefore, MIA or MIA2 might suppress T lymphocyte chemotaxis by masking fibronectin. TANGO expression is observed in many adult tissues [3]; we also confirmed weakly expression of TANGO in cancer-adjacent tissues (data not shown). In addition, we previously found that TANGO promotes tumor angiogenesis and lymphangiogenesis by activating PDGFB and neuropilin 2 [20]. Although TANGO is a ligand for CD11c/CD18 [5], we did not observe a direct interaction between TANGO and this receptor in OSCC cells [20]. More detailed studies will be needed to identify alternate receptors for TANGO in tumor cells; these might include other integrins or adhesion molecules.

In conclusion, we have demonstrated the utility of *MIA* gene family members as tumor markers, using a wide range of esophageal, lung, and cervical cancer samples. Although innumerable studies have investigated tumor biomarkers, the usefulness of molecular biomarkers for malignant tumors remains controversial. As *MIA* gene family members are secretory proteins [3], they might be detectable in serum, saliva, urine, ascites, pleural fluid, and other samples that can be collected more easily than tumors. Although additional detailed and large-scale examinations will be fundamental to determining the importance of *MIA* gene family members in cancers, our findings indicate that these proteins are alternative and efficacious diagnostic and treatment targets in human cancers. Our results therefore provide new knowledge about molecular tumor markers that could potentially improve the clinical outcomes and quality of life of affected patients.

## MATERIALS AND METHODS

### Tissue specimens

Randomly selected formalin-fixed, paraffin-embedded (FFPE) specimens were used for the present analysis. All FFPE samples were diagnosed at the Department of Molecular Pathology, Nara Medical University. To screen the expression of *MIA* family genes, we used a training cohort of 477 FFPE specimens in tumors of the following organs: 36 head and neck cancers, 15 salivary gland cancers, 10 esophageal cancers, 35 gastric cancers, 5 gastrointestinal tumors (GISTs) in the small intestine, 35 colorectal cancers, 16 liver cancers, 10 biliary cancers, 5 pancreatic cancers, 33 lung cancers, 33 bone and soft tissue malignant tumors, 3 skin malignant tumors, 19 breast cancers, 26 cervical cancers, 12 endometrial cancers, 47 ovarian cancers, 6 prostatic cancers, 11 testicular cancers, 17 bladder cancers, 16 kidney cancers, 15 thyroid cancers, 15 adrenal tumors, 20 malignant lymphomas, and 19 cancers of other tissues (Table 1).

Additionally, the validation set of *MIA* gene family expression comprised FFPE esophageal cancer, cervical cancer, and lung cancer tissues. The details of specimens are as follows: 66 cases of ESCC (49 men and 17 women, age range: 47–80 years, mean age = 67.5 years), 145 cases of lung cancer (107 men and 38 women, age range: 45–83 years, mean age = 66.7 years), and 126 cases of cervical cancer (age range: 24–74 years, mean age = 59.2 years). The lung cancer cases were subclassified as follows: 105 squamous cell carcinomas (SCCs), 25 adenocarcinomas, 10 small cell carcinomas, and 10 large cell neuroendocrine carcinomas (LCNECs). The 126 cervical cancers included 15 cases of cervical intraepithelial neoplasia 3 (CIN3) and 111 of invasive SCC. Among all cases used for the validation set of *MIA* gene family expression by immunohistochemistry, frozen and serum samples were available for gene expression and ELISA from cases with 10 ESCC, 14 lung cancer, and 13 cervical cancer, respectively. Each 5 samples of normal esophageal, lung, and cervical tissue and serum in healthy donor were used for control.

No patient received preoperative therapy. Tumor staging was performed according to the Union for International Cancer Control TNM classification system (seventh edition), and tumor histology was classified according to the World Health Organization criteria. Because written informed consent was not obtained for the immunohistochemical analysis, any identifying information was removed from the samples before the analysis to ensure the strict protection of patient privacy (unlinkable anonymizing). Written informed consent was obtained from individual patients for the use of their samples in the gene expression analysis and ELISA. The study plan was performed according to the ethical standards proposed in the *Declaration of Helsinki* and was approved by the Medical Ethics Committee of Nara Medical University, Kashihara, Japan (approval number. 719).

### Immunohistochemistry

Consecutive 3-μm sections were cut from each block and subjected to immunohistochemical staining with the EnVision+ DualLink system (DAKO, Carpinteria, CA, USA). After a 20-min antigen retrieval treatment with pepsin (DAKO), the sections underwent staining using an immunoperoxidase technique. Briefly, after a 15-min endogenous peroxidase block with 3% H_2_O_2_-methanol, specimens were incubated in a 10% skim milk solution (Morinaga Milk, Tokyo, Japan) for 20 min to block non-specific antibody reactions and rinsed 3 times with phosphate-buffered saline (PBS) (Sigma-Aldrich, St. Louis, MO, USA). Anti-MIA (Santa Cruz Biotechnology, Santa Cruz, CA, USA), anti-MIA2 (Abcam, Tokyo, Japan), and anti-TANGO/MIA3 antibodies (LifeSpan, Seattle, WA, USA) were diluted to 1 μg/ml and used as primary antibodies; after a two hour primary antibody incubation, the sections were incubated with a secondary antibody for 30 minutes at room temperature. The specimens were subsequently rinsed three times with PBS and color-developed using a diaminobenzidine (DAB) solution (DAKO). After washing to remove excess DAB solution, the specimens were counterstained with Meyer's hematoxylin (Sakura Finetek Japan, Tokyo, Japan). All samples were immunostained under the same antibody reaction and DAB exposure conditions. Appropriate negative and positive control slides were used.

### Evaluation of immunohistochemistry

MIA, MIA2, and TANGO immunoreactivities were classified according to the Allred's score (AS) [32]. We divided immunoreactivities into four grades based on AS: Grade 0, AS = 0; Grade 1, AS = 2–4; Grade 2, AS = 5–6; and Grade 3, AS = 7–8. Grade 2 and 3 cases were considered immunologically positive, in accordance with our previous report [33].

### RNA extraction and qRT-PCR

Total RNA was extracted using TRIzol reagent (Invitrogen, Carlsbad, CA, USA), and 1 mg of total RNA was converted to cDNA with a ReverTra Ace qPCR RT Kit (Toyobo, Osaka, Japan). Real-time RT- PCR was performed on a StepOnePlus Real-Time PCR System (Applied Biosystems, Foster City, CA, USA) using TaqMan Fast Universal PCR Master Mix (Applied Biosystems), and analyzed using the relative standard curve quantification method. The PCR conditions used were selected according to the manufacturer's manual and *glyceraldehyde-3-phosphate dehydrogenase* (*GAPDH*) mRNA was amplified as an internal control. TaqMan Gene Expression Assays of *MIA*(Hs00197954_m1), *MIA2* (Hs00365015_m1), *MIA3* (*TANGO*) (Hs00412706_m1), and *GAPDH* (ID: Hs03929097_g1) were purchased from Applied Biosystems. All PCRs were performed in triplicate.

### ELISA for MIA

The serum samples were obtained before treatment and stored at −80°C. Serum levels of MIA were measured by ELISA system for MIA (Roche Diagnostics, Mannheim, Germany) according to the manufacturer's instructions. All samples were tested in triplicate.

### Statistical analysis

Relationships between *MIA* gene family expression and clinicopathological parameters were calculated using the chi-square test or Fisher's exact test. Disease free survival was analyzed according to the Kaplan–Meier method, and differences between groups were calculated using a log-rank test. JMP8 software (SAS Institute, Cary, NC, USA) was used for all statistical analyses. *P* values < 0.05 were considered statistically significant.

## References

[1] Torre LA, Bray F, Siegel RL, Ferlay J, Lortet-Tieulent J, Jemal A (2015). Global cancer statistics, 2012. CA Cancer J Clin.

[2] Jia Y, Wang N, Wang J, Tian H, Ma W, Wang K, Tan B, Zhang G, Yang S, Bai B, Cheng Y (2014). Down-regulation of stromal caveolin-1 expression in esophageal squamous cell carcinoma: a potent predictor of lymph node metastases, early tumor recurrence, and poor prognosis. Ann Surg Oncol.

[3] Bosserhoff AK, Moser M, Buettner R (2004). Characterization and expression pattern of the novel MIA homolog TANGO. Gene expression patterns: GEP.

[4] Arndt S, Bosserhoff AK (2006). TANGO is a tumor suppressor of malignant melanoma. Int J Cancer.

[5] Arndt S, Melle C, Mondal K, Klein G, von Eggeling F, Bosserhoff AK (2007). Interactions of TANGO and leukocyte integrin CD11c/CD18 regulate the migration of human monocytes. J Leukocyte Biol.

[6] Arndt S, Bosserhoff AK (2007). Reduced expression of TANGO in colon and hepatocellular carcinomas. Oncol Rep.

[7] Bosserhoff AK, Buettner R (2002). Expression, function and clinical relevance of MIA (melanoma inhibitory activity). Histol Histopathol.

[8] Bosserhoff AK, Kaufmann M, Kaluza B, Bartke I, Zirngibl H, Hein R, Stolz W, Buettner R (1997). Melanoma-inhibiting activity, a novel serum marker for progression of malignant melanoma. Cancer Res.

[9] Bosserhoff AK, Stoll R, Sleeman JP, Bataille F, Buettner R, Holak TA (2003). Active detachment involves inhibition of cell-matrix contacts of malignant melanoma cells by secretion of melanoma inhibitory activity. Lab Invest.

[10] El Fitori J, Kleeff J, Giese NA, Guweidhi A, Bosserhoff AK, Buchler MW, Friess H (2005). Melanoma Inhibitory Activity (MIA) increases the invasiveness of pancreatic cancer cells. Cancer cell international.

[11] Aung PP, Oue N, Mitani Y, Nakayama H, Yoshida K, Noguchi T, Bosserhoff AK, Yasui W (2006). Systematic search for gastric cancer-specific genes based on SAGE data: melanoma inhibitory activity and matrix metalloproteinase-10 are novel prognostic factors in patients with gastric cancer. Oncogene.

[12] Winklmeier A, Contreras-Shannon V, Arndt S, Melle C, Bosserhoff AK (2009). Cadherin-7 interacts with melanoma inhibitory activity protein and negatively modulates melanoma cell migration. Cancer Sci.

[13] Bosserhoff AK, Moser M, Scholmerich J, Buettner R, Hellerbrand C (2003). Specific expression and regulation of the new melanoma inhibitory activity-related gene MIA2 in hepatocytes. J Biol Chem.

[14] Hellerbrand C, Bataille F, Schlegel J, Hartmann A, Muhlbauer M, Scholmerich J, Buttner R, Hofstadter F, Bosserhoff AK (2005). *In situ* expression patterns of melanoma inhibitory activity 2 in healthy and diseased livers. Liver international.

[15] Hellerbrand C, Amann T, Schlegel J, Wild P, Bataille F, Spruss T, Hartmann A, Bosserhoff AK (2008). The novel gene MIA2 acts as a tumour suppressor in hepatocellular carcinoma. Gut.

[16] Kong B, Wu W, Valkovska N, Jager C, Hong X, Nitsche U, Friess H, Esposito I, Erkan M, Kleeff J, Michalski CW (2015). A common genetic variation of melanoma inhibitory activity-2 labels a subtype of pancreatic adenocarcinoma with high endoplasmic reticulum stress levels. Scientific reports.

[17] Sasahira T, Kirita T, Oue N, Bhawal UK, Yamamoto K, Fujii K, Ohmori H, Luo Y, Yasui W, Bosserhoff AK, Kuniyasu H (2008). High mobility group box-1-inducible melanoma inhibitory activity is associated with nodal metastasis and lymphangiogenesis in oral squamous cell carcinoma. Cancer Sci.

[18] Sasahira T, Kirita T, Kurihara M, Yamamoto K, Bhawal UK, Bosserhoff AK, Kuniyasu H (2010). MIA-dependent angiogenesis and lymphangiogenesis are closely associated with progression, nodal metastasis and poor prognosis in tongue squamous cell carcinoma. Eur J Cancer.

[19] Kurihara M, Kirita T, Sasahira T, Ohmori H, Matsushima S, Yamamoto K, Bosserhoff AK, Kuniyasu H (2013). Protumoral roles of melanoma inhibitory activity 2 in oral squamous cell carcinoma. Br J Cancer.

[20] Sasahira T, Kirita T, Yamamoto K, Ueda N, Kurihara M, Matsushima S, Bhawal UK, Bosserhoff AK, Kuniyasu H (2014). Transport and Golgi organisation protein 1 is a novel tumour progressive factor in oral squamous cell carcinoma. Eur J Cancer.

[21] Biomarkers Definitions Working G (2001). Biomarkers, surrogate endpoints: preferred definitions and conceptual framework. Clin Pharmacol Ther.

[22] Sasahira T, Kirita T, Kuniyasu H (2014). Update of molecular pathobiology in oral cancer: a review. International journal of clinical oncology.

[23] Wu JY, Yi C, Chung HR, Wang DJ, Chang WC, Lee SY, Lin CT, Yang YC, Yang WC (2010). Potential biomarkers in saliva for oral squamous cell carcinoma. Oral Oncol.

[24] Ikota H, Nobusawa S, Tanaka Y, Yokoo H, Nakazato Y (2011). High-throughput immunohistochemical profiling of primary brain tumors and non-neoplastic systemic organs with a specific antibody against the mutant isocitrate dehydrogenase 1 R132H protein. Brain Tumor Pathol.

[25] Jachimczak P, Apfel R, Bosserhoff AK, Fabel K, Hau P, Tschertner I, Wise P, Schlingensiepen KH, Schuler-Thurner B, Bogdahn U (2005). Inhibition of immunosuppressive effects of melanoma-inhibiting activity (MIA) by antisense techniques. Int J Cancer.

[26] Bauer R, Humphries M, Fassler R, Winklmeier A, Craig SE, Bosserhoff AK (2006). Regulation of integrin activity by MIA. J Biol Chem.

[27] Graf SA, Busch C, Bosserhoff AK, Besch R, Berking C (2014). SOX10 promotes melanoma cell invasion by regulating melanoma inhibitory activity. J Invest Dermatol.

[28] Bosserhoff AK, Moser M, Hein R, Landthaler M, Buettner R (1999). *In situ* expression patterns of melanoma-inhibiting activity (MIA) in melanomas and breast cancers. J Pathol.

[29] Bosserhoff AK, Kondo S, Moser M, Dietz UH, Copeland NG, Gilbert DJ, Jenkins NA, Buettner R, Sandell LJ (1997). Mouse CD-RAP/MIA gene: structure, chromosomal localization, and expression in cartilage and chondrosarcoma. Dev Dyn.

[30] Hau P, Ruemmele P, Kunz-Schughart LA, Doerfelt A, Hirschmann B, Lohmeier A, Koch H, Mueller A, Bogdahn U, Bosserhoff AK (2004). Expression levels of melanoma inhibitory activity correlate with time to progression in patients with high-grade glioma. Oncol Rep.

[31] Savino W, Mendes-da-Cruz DA, Silva JS, Dardenne M, Cotta-de-Almeida V (2002). Intrathymic T-cell migration: a combinatorial interplay of extracellular matrix and chemokines?. Trends Immunol.

[32] Allred DC, Harvey JM, Berardo M, Clark GM (1998). Prognostic and predictive factors in breast cancer by immunohistochemical analysis. Mod Pathol.

[33] Sasahira T, Ueda N, Yamamoto K, Kurihara M, Matsushima S, Bhawal UK, Kirita T, Kuniyasu H (2014). Prox1 and FOXC2 act as regulators of lymphangiogenesis and angiogenesis in oral squamous cell carcinoma. PLoS ONE.

